# Neuronal Activity Patterns Regulate Brain-Derived Neurotrophic Factor Expression in Cortical Cells *via* Neuronal Circuits

**DOI:** 10.3389/fnins.2021.699583

**Published:** 2021-12-10

**Authors:** Yumi Miyasaka, Nobuhiko Yamamoto

**Affiliations:** Laboratory of Cellular and Molecular Neurobiology, Graduate School of Frontier Biosciences, Osaka University, Suita, Japan

**Keywords:** activity dependence, promoter activity, gene expression, BDNF, cortex, organotypic culture, live imaging, calcium signaling

## Abstract

During development, cortical circuits are remodeled by spontaneous and sensory-evoked activity *via* alteration of the expression of wiring molecules. An intriguing question is how physiological neuronal activity modifies the expression of these molecules in developing cortical networks. Here, we addressed this issue, focusing on brain-derived neurotrophic factor (BDNF), one of the factors underlying cortical wiring. Real-time imaging of BDNF promoter activity in organotypic slice cultures revealed that patterned stimuli differentially regulated the increase and the time course of the promoter activity in upper layer neurons. Calcium imaging further demonstrated that stimulus-dependent increases in the promoter activity were roughly proportional to the increase in intracellular Ca^2+^ concentration per unit time. Finally, optogenetic stimulation showed that the promoter activity was increased efficiently by patterned stimulation in defined cortical circuits. These results suggest that physiological stimulation patterns differentially tune activity-dependent gene expression in developing cortical neurons *via* cortical circuits, synaptic responses, and alteration of intracellular calcium signaling.

## Introduction

Neuronal activity plays a crucial role in the formation of functional connections during development. This activity-dependent circuit formation has been well characterized in the sensory cortex: Spontaneous and sensory-evoked neuronal activity remodels neuronal connections in the visual and somatosensory cortex (Katz and Shatz, 1996; Feldman and Brecht, 2005; Hensch, 2005; Ackman and Crair, 2014). Moreover, such remodeling is regulated by activity-dependent expression of molecules that affect axon and dendrite behavior, indicating that neuronal activity is adequately converted into molecular signals (Iwasato et al., 2000; Uesaka et al., 2005, 2007; Mizuno et al., 2007; Yamada et al., 2010; Mire et al., 2012; Hayano et al., 2014; Munz et al., 2014; Suárez et al., 2014; Nakashima et al., 2019).

The next challenge is to understand how physiological neuronal activity patterns affect the gene expression that regulates cortical wiring. Indeed, neuronal firing is known to occur with characteristic frequency *in vivo* during cortical development (Minlebaev et al., 2007; Yang et al., 2009; Luhmann and Khazipov, 2018). For example, long-beta oscillation and gamma rhythmic activity are prominently generated spontaneously or in a stimuli-evoked manner during the early postnatal period. Theses characteristic neural activities are thought to contribute to circuit remodeling, but few studies have investigated how patterned activity influences gene expression (Lee et al., 2017; Tyssowski et al., 2018).

To address this issue, we focused on brain-derived neurotrophic factor (BDNF), one of the best characterized activity-dependent molecules, which is expressed in upper layer neurons (Castren et al., 1992; Rocamora et al., 1996; Majdan and Shatz, 2006) and affects cortical wiring by promoting axonal and dendritic growth (Cabelli et al., 1995; McAllister et al., 1996; Horch and Katz, 2002; Wirth et al., 2003; Jeanneteau et al., 2010; Granseth et al., 2013; Park and Poo, 2013; Niculescu et al., 2018). Transcriptional regulation of the Bdnf gene, which responds to neuronal activity, has also been studied biochemically (West et al., 2002; Flavell and Greenberg, 2008; Greer and Greenberg, 2008; Zheng et al., 2012; Madabhushi and Kim, 2018). Fundamentally, Ca^2+^ influx triggers activation of calcium-dependent transcription factors such as cAMP response element binding protein (CREB), and their binding to specific DNA sites including BDNF promoter induces the expression of downstream genes (Shieh et al., 1998; Tao et al., 1998, 2002; West et al., 2001; Chen et al., 2003; Pfenning et al., 2010). Such characterization allows us to study the activity-dependent gene regulation mechanism from the physiological and molecular biological points of view.

In the present study, we investigated how neuronal activity patterns modify Bdnf promoter activity in living individual cortical neurons. For real-time imaging of the promoter activity, a luciferase expression vector under the control of the Bdnf exon IV promoter, which is the best characterized promoter in the activity-dependent context, was electroporated into upper layer neurons in organotypic cortical cultures. In this culture, cortical cytoarchitecture is preserved, and intracortical connections such as horizontal connections that connect upper layer neurons are formed in an activity-dependent fashion, as they are *in vivo* (Gilbert and Wiesel, 1989; Callaway and Katz, 1990; Yamamoto et al., 1992; Ruthazer and Stryker, 1996; Uesaka et al., 2005; Ohnami et al., 2008). BDNF promoter activity as well as calcium dynamics were then investigated in upper layer neurons by stimulating the cortical circuits electrophysiologically and optogenetically with various stimulus patterns. The results demonstrated that patterned neuronal activity differentially modulates Bdnf expression *via* alteration of the calcium signaling within the cortical networks.

## Results

### Brain-Derived Neurotrophic Factor Promoter Activity Changes Temporally in Individual Cortical Cells in Response to Chemical Stimulation

**FIGURE 1:**
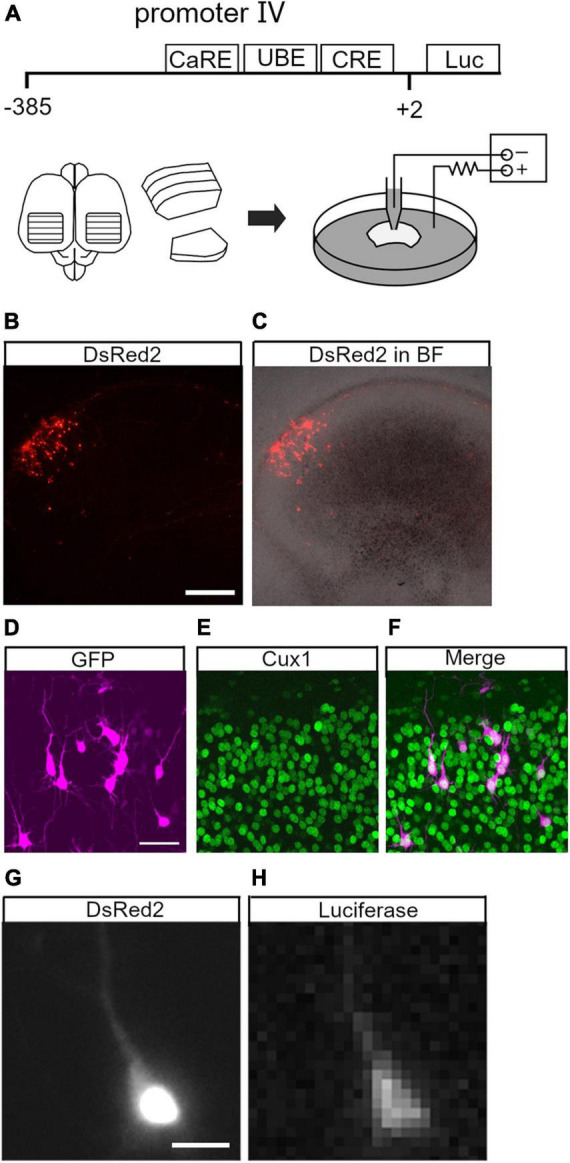
Live imaging of brain-derived neurotrophic factor (BDNF) promoter activity in upper layer neurons. **(A)** Brain-derived neurotrophic factor promoter region and schematic representation of organotypic cortical slice culture and electroporation. CaRE, calcium response element; UBE, upstream stimulatory factor binding element; CRE, cAMP-response element; Luc, luciferase. **(B,C)** Electroporated (DsRed2-labeled) cells in a cortical slice. Fluorescence image **(B)** and merged image with bright field (BF) **(C)**. **(D–F)** Cux1 immunoreactivity in the electroporated cells. Electroporated neurons **(D)** with immunostaining for Cux1 **(E)** and merged image **(F)**. **(G,H)** Luciferase signal in an electroporated neuron. The neuron exhibits DsRed2 **(G)** and luciferase signals **(H)**. Scale bars: **(B)** 500 μm; **(D)** 50 μm; **(G)** 20 μm.

To study neuronal activity-dependent Bdnf promoter activity in individual cortical neurons, we performed live imaging with a luciferase assay in organotypic rat cortical cultures (Yamamoto et al., 1992). The promoter activity was investigated in upper layer neurons, as they have BDNF-dependent plasticity in terms of development and circuit formation (McAllister et al., 1996; Horch et al., 1999; Niblock et al., 2000; Granseth et al., 2013). A luciferase expression plasmid containing Bdnf promoter IV (Bdnf-luc plasmid), which primarily underlies activity-dependent Bdnf expression (Tao et al., 1998; Aid et al., 2007; Hong et al., 2008; Sakata et al., 2009), was used for live imaging (Figure 1A). The promoter region is located immediately upstream of the transcription initiation site and is highly conserved among species (Supplementary Figure 1; Pruunsild et al., 2011). The Bdnf-luc plasmid was electroporated sparsely into upper layer cells (Figure 1A; Uesaka et al., 2005). Enhanced green fluorescent protein (EGFP)- or DsRed2-encoding plasmid was co-transfected to identify electroporated cells readily and to reveal cellular morphology. As shown in Figures 1B,C, fluorescent protein-labeled cells were distributed in the upper layers (0.1–0.5 mm from the pial surface) of cortical explants. Immunohistochemistry showed that the transfected cells were mostly Cux1-immunopositive (85%, 156/183 cells from 11 slices) (Figures 1D–F). Roughly half of the fluorescent protein-labeled cells exhibited bioluminescent signals in the presence of luciferin (Figures 1G,H).

**FIGURE 2:**
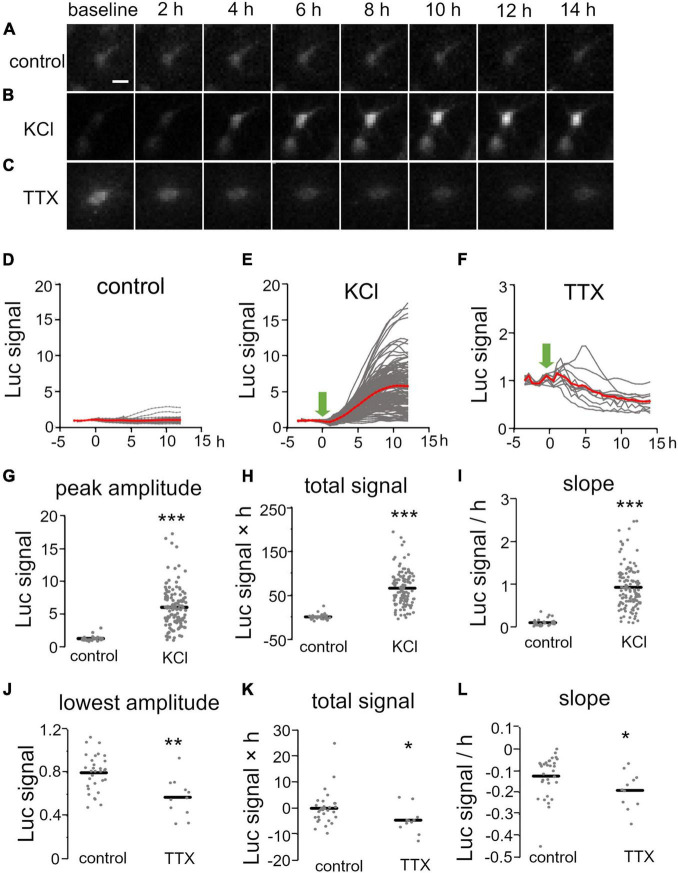
Temporal properties of Bdnf promoter activity in response to pharmacological stimulation. **(A–C)** Time lapse images of luciferase signals without treatment **(A)** and with KCl **(B)** and TTX **(C)** treatment. Each picture was taken at the indicated time. Scale bar, 20 μm. **(D–F)** Time courses of luciferase signals in individual neurons without treatment **(D)** and with KCl **(E)** and TTX **(F)** treatment. Gray lines indicate luciferase signals in each neuron, and red lines show averages. Arrows indicate the initiation of the treatment. **(G–L)** Quantitative analysis of KCl **(G–I)** and TTX treatment **(J–L)**. The peak amplitude **(G)**, lowest amplitude **(J)**, total signals **(H,K)**, and slopes **(I,L)** were analyzed. Horizontal bars represent the average values. Asterisks indicate a significant difference compared to control (Mann–Whitney *U* test, *P < 0.05, **P < 0.01, ***P < 0.001).

We then investigated temporal changes of Bdnf promoter activity using KCl treatment (final concentration, 25 mM) at 1 week *in vitro*, when spontaneous neuronal activity is very low (Uesaka et al., 2005). Endogenous Bdnf expression in cortical explants was confirmed to increase markedly upon treatment (Supplementary Figure 2A). For quantification of the promoter activity in individual cells, the bioluminescent signals were normalized by the baseline intensity in each cell and are referred to here as “luciferase signals” (see “Materials and Methods”). In the absence of KCl, the luciferase signals in individual cells were almost unchanged for up to 20 h (Figures 2A,D), but the signals in most cells started to increase within 2 h after the initiation of KCl treatment, and reached a maximal value after approximately 10 h (Figures 2B,E and Supplementary Movie1). To characterize the increase in Bdnf promoter activity, we measured the peak amplitude, total signal, and slope of the luciferase signals, which reflect the maximum level, gross amount, and rapidity of Bdnf expression, respectively (see “Materials and Methods”). These parameters were considerably larger in KCl-treated than in untreated cultures (Figures 2G–I and Table 1). Interestingly, the increase in Bdnf promoter activity varied widely among cells (Figures 2E,G). The peak amplitude was not related to the baseline intensity (Supplementary Figure 3), suggesting that the luciferase signals in individual cells are not due to transfection efficiency, but reflect their own expression levels.

**TABLE 1:** Brain-derived neurotrophic factor (BNDF) promoter activity in response to pharmacological treatment.

Treatment	Cell #	Slice #	Peak amplitude (fold change)	Lowest amplitude (fold change)	Total signal (fold change × time [h])	Slope (positive)(fold change/h)	Slope (negative)(fold change/h)
KCl	123	9	6.1 ± 0.28***	–	65± 3.5***	0.92 ± 0.046***	–
TTX	12	2	–	0.57 ± 0.054**	−4.6 ± 1.5*	–	−0.19 ± 0.025*
Control	31	5	1.3 ± 0.070	0.80 ± 0.030	−0.064 ± 1.1	0.099 ± 0.015	−0.12 ± 0.017

Asterisks indicate significant change compared to control (Mann-Whitney U test, *p < 0.05, **p < 0.01, ***p < 0.001).

*Slope (positive) and slope (negative) indicate the maximal inclination of the positive and negative curves, respectively.*

The effect of neuronal activity on Bdnf promoter activity was also examined by suppressing neuronal firing at 2 weeks *in vitro*, when spontaneous activity is prominent (see below) (Uesaka et al., 2005). To inhibit spontaneous firing activity, a sodium channel blocker, tetrodotoxin (TTX), was applied to the culture medium (final concentration, 100 nM). In accordance with a considerable decrease in endogenous Bdnf expression (Supplementary Figure 2B), the luciferase signal gradually decreased and reached its lowest amplitude approximately 10 h after TTX addition (Figures 2C,F,J–L and Table 1). Thus, pharmacological treatments demonstrated that Bdnf promoter activity was temporally and differentially regulated in individual cortical neurons by neuronal activity.

As described above, Bdnf promoter activity differed considerably among cells (Figure 2E). We investigated whether this individual variability is related to the spatial arrangement of cortical neurons. We found that cells with similar peak amplitude of the luciferase signal after KCl treatment tended to cluster (Supplementary Figure 4A). Quantitative analysis showed that the proportion of “similar pairs,” cell pairs with similar promoter activity, was significantly higher (*P < 0.05, ^**^P < 0.01, Fisher’s Exact Test) in cell pairs that were located close to each other (intercellular distances < 30 μm) than in more widely separated cell pairs (intracellular distances > 30 μm) (Supplementary Figures 4B–D, Supplementary Table 1, and see “Materials and Methods”). These findings suggest that cortical neurons with similar activity dependence on Bdnf expression are located in close proximity to each other under the uniform depolarization of KCl treatment.

### Patterned Stimuli Differentially Alter Brain-Derived Neurotrophic Factor Promoter Activity

Next, we investigated how patterned neuronal activity influences Bdnf promoter activity. As Bdnf expression in physiological conditions is regulated postsynaptically by excitatory synaptic inputs (Castren et al., 1992; Rocamora et al., 1996; Zheng et al., 2011), the promoter activity was examined by stimulating inputs after 2 weeks in culture, when intrinsic cortical connections are established with synapse formation (Yamamoto et al., 1992; Uesaka et al., 2005; Matsumoto et al., 2016). To clarify the effects of the excitatory inputs, inhibitory transmission was suppressed by adding a GABAA receptor blocker, picrotoxin (PTX), to the culture medium. However, a complication was the occurrence of frequent spontaneous firing activity at this stage (Supplementary Figure 5; Uesaka et al., 2005), which interferes with the assessment of the role of evoked activity on the promoter activity. To reduce the spontaneous activity without perturbing synaptic responses, the concentrations of Ca^2+^ and Mg^2+^ in the extracellular medium were raised (Gustafsson et al., 1987). Under this condition, spontaneous firing activity was almost abolished (Supplementary Figure 5) without diminishing activity-dependent transcription (Supplementary Figure 6 and Supplementary Table 2).

Conventional electrical stimulation was carried out with a pair of platinum wires that were embedded into culture dishes. In our culture system, intrinsic cortical connections including horizontal connections are preserved (Uesaka et al., 2005). To elicit the synaptic inputs to BDNF-luc transfected cells, these electrodes penetrated the cortical slice away from the Bdnf-luc-transfected region to avoid antidromic activation (approximately 1 mm away from recorded cells, Supplementary Figure 7A). Calcium imaging with GCaMP6f or Oregon Green 488 BAPTA-1 (OGB-1) showed that upper layer neurons faithfully responded to each stimulation (Supplementary Figure 7B and Supplementary Movie 2). These responses were almost abolished with DNQX and D-AP5 (Supplementary Figure 7B), indicating that evoked responses were largely due to excitatory synaptic activation.

**FIGURE 3:**
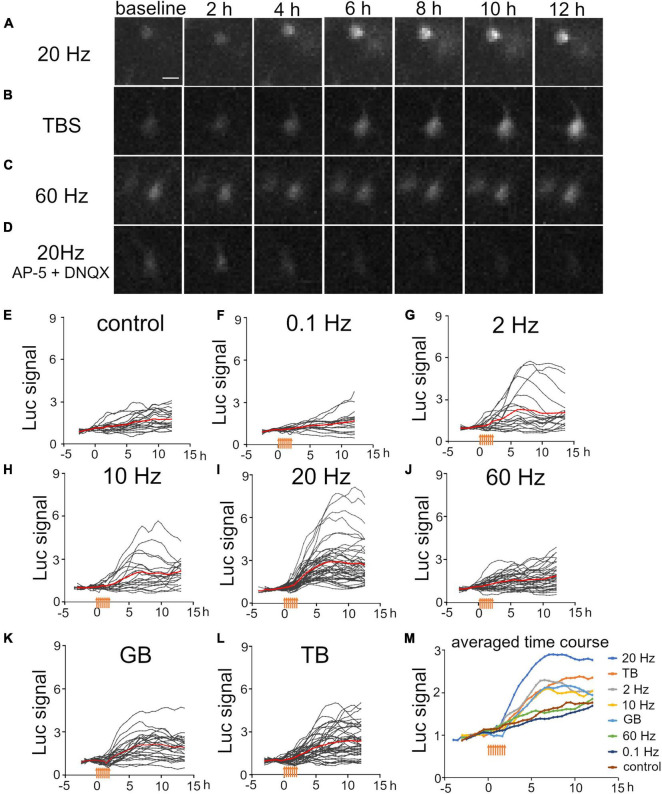
Temporal properties of Bdnf promoter activity in response to electrical stimulation at various frequencies. **(A–D)** Time lapse images of luciferase signals for 20 Hz stimulation **(A)**, TB stimulation **(B)**, 60 Hz stimulation **(C)**, and 20 Hz stimulation in the presence of AP-5 and DNQX **(D)**. Each picture was taken at the indicated time. Scale bar, 20 μm. **(E–L)** Time courses of luciferase signals in individual neurons for control **(E)**, 0.1 Hz **(F)**, 2 Hz **(G)**, 10 Hz **(H)**, 20 Hz **(I)**, 60 Hz **(J)**, GB **(K)**, and TB **(L)** stimulation. Gray lines indicate luciferase signals in each neuron, and red lines show the averages. **(M)** Averaged time courses of the luciferase signals for each stimulation. The signals were averaged from all cortical neurons shown in panels **(E–L)**. Orange arrows indicate stimulus time points.

It is known that characteristic neuronal activities are generated in the developing brain (Yang et al., 2009; Kilb et al., 2011; Luhmann and Khazipov, 2018). To mimic the occurrence of such neuronal activities, we applied constant-frequency stimulation (0.1 and 2 Hz for delta band, 10 Hz for alpha band, 20 Hz for beta band, and 60 Hz for gamma band) and burst stimulation (60-Hz burst at 0.33 Hz for gamma burst and 100-Hz burst at 5 Hz for theta burst). Luciferase signals were then measured in individual upper layer neurons. We found that some patterned stimuli clearly increased the luciferase signals, but others seemed to be ineffective (Figures 3A–C). Indeed, the time courses of the luciferase signals showed that Bdnf promoter activity increased differently by the stimulation patterns (Figures 3E–M). Moreover, as was the case with KCl treatment, not all transfected cells exhibited positive responses (Figures 3E–L). These responses were confirmed to be evoked synaptically, since the stimulation did not increase the luciferase signals in the presence of synaptic blockers (Figure 3D).

**FIGURE 4:**
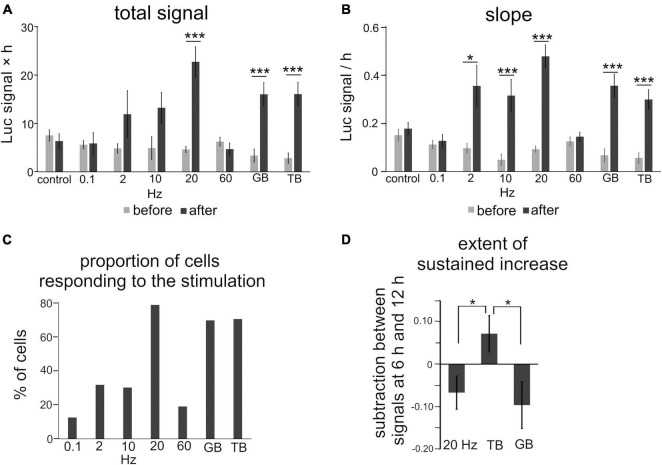
Quantitative analysis of the effects of patterned electrical stimulation. **(A,B)** Increased levels for total signals **(A)** and slope **(B)**. **(C)** Proportion of cells responding to each stimulation. **(D)** Extent of the sustained increase for 20 Hz, GB, and TB stimulation. Asterisks indicate a significant difference between the signals before and after stimulation **(A,B)** or among 20 Hz, GB, and TB stimulation **(D)** (Mann–Whitney *U* test or Tukey’s multiple comparison test, *P < 0.05, ***P < 0.001). Bars represent the mean ± SEM.

Luciferase signals tended to increase slightly and gradually even without stimulation, probably due to removal of inhibitory transmission (Figure 3E). To quantify the effects of each stimulation pattern, the total signals and slopes in Bdnf promoter activity were compared before and after stimulation. We found that stimuli with several frequency patterns were effective in increasing the promoter activity (Figures 4A,B). The total signals were 2- to 4-fold larger in average after applying 2, 10, 20 Hz, GB, and TB stimulation although the difference was not significant in 2 Hz and 10 Hz stimulation (Table 2). The slopes were 3- to 6-fold larger after 2, 10, 20 Hz, GB, and TB. In contrast, 0.1 and 60 Hz stimulation elevated neither the total signals nor the slopes (Figures 4A,B and Table 2). Next, the proportion of cells that responded to the electrical stimulation was examined. Stimulation with 20 Hz, GB, and TB activated roughly 70% of the analyzed cells, but the other stimulation patterns activated only less than 35% of the cells (Figure 4C). These results indicate that different patterned activity differentially regulated the transcriptional activity.

**TABLE 2:** Brain-derived neurotrophic factor promoter activity in response to patterned electrical stimulation in rat cortical neurons.

Stimulation pattern	Cell #	Slice #	Before or after stimulation	Total signal	Slope
				(fold change	(fold change/
				× time [h])	time [h])
					
Control	21	5	Before	7.5 ± 1.2	0.15 ± 0.023
			After	6.3 ± 1.5	0.18 ± 0.026
0.1 Hz	16	2	Before	5.6 ± 0.86	0.11 ± 0.017
			After	5.9 ± 2.3	0.13 ± 0.027
2 Hz	19	3	Before	6.4 ± 1.3	0.097 ± 0.019
			After	12 ± 4.9	0.35 ± 0.086*
10 Hz	20	5	Before	3.6 ± 1.1	0.049 ± 0.024
			After	13 ± 3.2	0.31 ± 0.067***
20 Hz	46	4	Before	5.6 ± 0.91	0.092 ± 0.013
			After	23 ± 3.1***	0.48 ± 0.048***
60 Hz	37	3	Before	7.6 ± 1.2	0.12 ± 0.018
			After	4.7 ± 1.3	0.14 ± 0.019
GB	26	3	Before	3.4 ± 1.3	0.067 ± 0.027
			After	16 ± 2.4***	0.35 ± 0.049***
TB	40	4	Before	6.4 ± 1.3	0.056 ± 0.021
			After	16 ± 2.4***	0.30 ± 0.041***

Asterisks indicate significant increase compared to before for total signal and slope, and compared to TB for extent of sustained increase (Mann-Whitney U test, *p < 0.05, ***p < 0.001).

Regarding the time course, the luciferase signals started to increase in every stimulation pattern within 2 h after the initiation of stimulation, but the profiles of time courses varied depending on the stimulation pattern (Figure 3M). We analyzed the time courses of luciferase signals in response to 20 Hz, TB, and GB stimulation, which were most effective in inducing the promoter activity (Figures 4A–C). In the cases of 20 Hz and GB stimulation, the signals mostly peaked around 6 h after stimulation, and then plateaued or slightly decreased (Figures 3I,K,M). In contrast, TB stimulation tended to increase the signals in a gradual fashion until the end of the experiment session (Figures 3L,M). Quantitative analysis of the time courses showed that the extent of the sustained increase was significantly larger (−0.067 ± 0.039 for 20 Hz, −0.096 ± 0.055 for GB, 0.072 ± 0.043* for TB, Tukey’s multiple comparison test, *P < 0.05) in TB stimulation (Figure 4D), suggesting that the time course of Bdnf promoter activity is also modulated by the stimulation patterns.

### Stimulus-Dependent Increase in Brain-Derived Neurotrophic Factor Promoter Activity Is Related to Transient Changes of Intracellular Ca^2+^ Concentration

**FIGURE 5:**
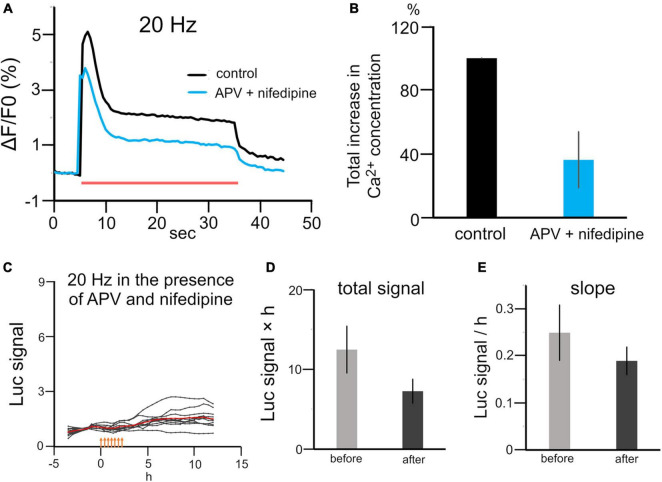
Effects of calcium channel blockers on stimulation-induced luciferase expression. **(A)** Calcium signaling during 20 Hz stimulation in the presence or absence of APV and nifedipine. A red bar represents the period of stimulation. **(B)** Quantitative analysis of the intracellular Ca^2+^ concentration with and without APV and nifedipine (*n* = 3). **(C)** Time courses of luciferase signals with 20 Hz stimulation in the presence of APV and nifedipine. Orange arrows indicate stimulus time points. **(D,E)** Changes in the total signal **(D)** and slope **(E)** in the presence of APV and nifedipine.

To investigate how patterned stimuli differentially regulated Bdnf promoter activity, we investigated intracellular Ca^2+^ levels in each stimulation pattern, because previous studies have shown that Bdnf promoter activity is regulated by calcium-dependent mechanisms (West et al., 2002). Calcium imaging with OGB-1 showed that the intracellular Ca^2+^ concentrations were significantly suppressed during 20 Hz stimulation in the presence of nifedipine and APV, blockers of L-type voltage-gated Ca^2+^ channel and NMDA channel, which are the main source of Ca^2+^ influx (approximately 50% reduction, *n* = 3 cultures, Figures 5A,B). In this condition, 20 Hz stimulation, which is the most effective to increase the promoter activity, did not increase the total signal or the slope after stimulation (total signal, 7.3 ± 1.5 vs 12 ± 2.9; slope, 0.19 ± 0.029 vs 0.25 ± 0.059, *n* = 11 cells from two cultures, *P* > 0.05, Mann–Whitney *U* test; Figures 5C–E). This result confirms that electrical stimulation up-regulated Bdnf promoter activity *via* intracellular calcium signaling.

**FIGURE 6:**
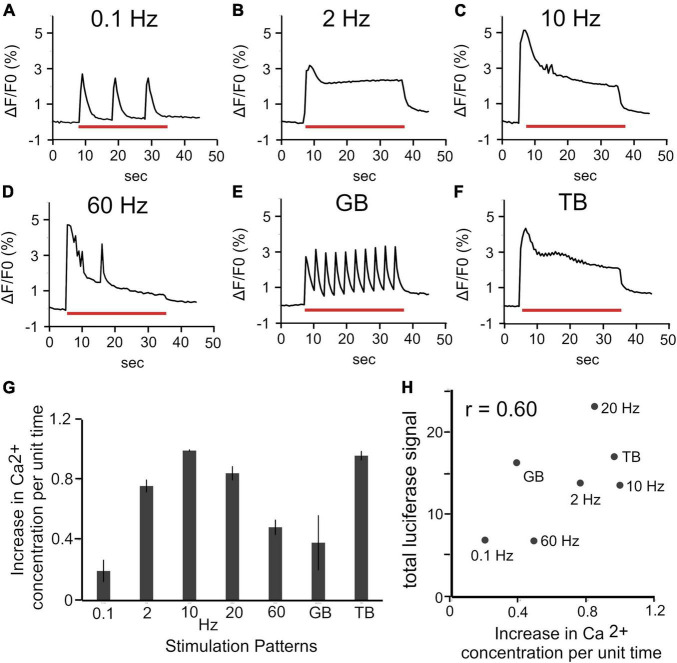
Relationship between intracellular Ca^2+^ concentration and luciferase signals. **(A–F)** Changes in intracellular Ca^2+^ concentration (ΔF/F0) in upper layers during various stimulations. Red bars represent the period of stimulation (30 s). **(G)** Increases in intracellular Ca^2+^ concentration per unit time for each stimulation pattern (*n* = 6 slices). In panel **(G)**, the value for each stimulation was calculated as the ratio to the maximum among the increases for all stimulation patterns in each slice, and averaged for the number of samples. **(H)** Relationship between total luciferase signals and changes in intracellular Ca^2+^ concentration per unit time.

We hypothesized that the different increases in Bdnf promoter activity in response to stimulation patterns are due to the distinct intracellular Ca^2+^ concentrations. To test this, the calcium responses to each patterned stimulation were measured with OGB-1. Calcium imaging showed that different stimulation patterns evoked different calcium oscillations (Figures 6A–F). The increased Ca^2+^ concentrations per unit time were also different among the stimulation patterns (see “Materials and Methods”). The increase levels per unit time (normalized by the maximal value) were 0.20 ± 0.071 for 0.1 Hz, 0.76 ± 0.039 for 2 Hz, 1.00 ± 0.0047 for 10 Hz, 0.85 ± 0.044 for 20 Hz, 0.49 ± 0.048 for 60 Hz, 0.38 ± 0.18 for GB, and 0.96 ± 0.028 for TB (Figure 6G). These values were plotted against the promoter activity obtained by the luciferase assay (Figure 4A) for each stimulation (Figure 6H). The total luciferase signals were highly correlated (*r* = 0.60, correlation coefficient) with intracellular Ca^2+^ concentrations per unit time (Figure 6H). Thus, it is likely that physiological patterned activities differentially regulate Bdnf promoter activity through altering intracellular Ca^2+^ concentrations.

### Brain-Derived Neurotrophic Factor Promoter Activity Is Induced in Cortical Neurons Postsynaptically *via* Upper Layer Neuronal Circuits

**FIGURE 7:**
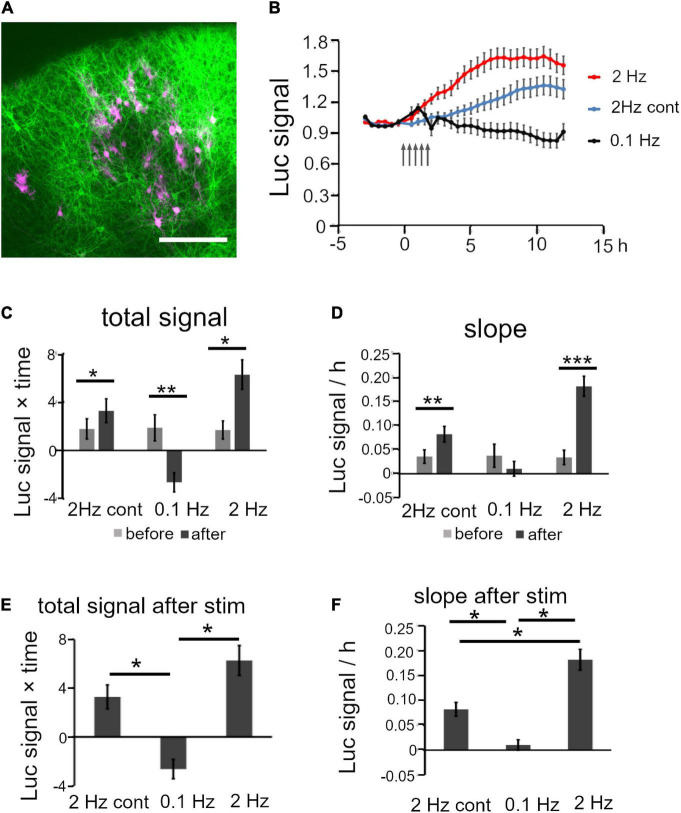
Analysis of Bdnf promoter activity in response to optogenetic stimulation. **(A)** Representative confocal image of a cortical slice electroporated with ChR2 and luciferase vectors (green, ChR2-positive and red, luciferase-positive cells). Scale bar, 200 μm. **(B)** Time courses of luciferase signals in each stimulation pattern. Arrows indicate the timing of stimulation. Bars represent SEM. **(C,D)** Quantitative analysis of the increased level of luciferase signals for total signals **(C)** and slope **(D)** before and after stimulation. **(E,F)** Comparison of the increase level of luciferase signals between stimulation patterns for total signal **(E)** and slope **(F)**. **(E)** and **(F)** are extracted from **(C)** and **(D)**, respectively. Bars represent SEM. Asterisks indicate a significant difference (Mann–Whitney *U* test or Turkey’s multiple comparison test, *P < 0.05, **P < 0.01, ***P < 0.001).

The electrical stimulation experiment demonstrated that BDNF promoter activity was regulated differently by stimulation patterns. An intriguing issue is whether physiological activity in defined cortical circuits influences gene expression. To examine this issue, we performed an optogenetic experiment focusing on intracortical connections between upper layer neurons (Gilbert and Wiesel, 1979; Yoshimura et al., 2005), in which the luciferase signals were recorded in upper layer neurons by stimulating other upper layer neurons. For this, a channel rhodopsin 2 (chr2) plasmid was transfected by *in utero* electroporation into mouse progenitor cells at E15 which are destined for the upper layers. After giving birth, cortical slices containing ChR2-expressing cells were cultured, and then the Bdnf-luc plasmid was transfected into upper layer cells (Figure 7A). Bdnf-luc-transfected cells in mice cortical explants were mostly Cux-1 positive, and were surrounded by ChR2-expressing fibers (Supplementary Figures 8A–F). As these ChR2-transfected cells were upper layer excitatory neurons (Supplementary Figures 8G–I), the optogenetic stimulation should produce excitatory synaptic responses in luciferase-expressing upper layer cells *via* the intracortical connections.

Whether the Bdnf promoter was activated similarly in mouse cortical neurons was confirmed prior to the optogenetic stimulation experiment. Pharmacological treatment showed that the promoter activity was remarkably increased by KCl treatment (Supplementary Figure 9A). The increased levels of Bdnf promoter activity were comparable with those in rat neurons (Supplementary Figures 9B–D and Supplementary Table 2). The increases and temporal properties of Bdnf promoter activity after patterned stimulation were also similar to those for rat neurons (Supplementary Figure 10 and Supplementary Table 3). Thus, neuronal activity modulates the promoter activity in a similar manner in both mice and rats.

As hChR2(H134R) did not tend to respond to high frequency stimulation (Cruikshank et al., 2010; Olsen et al., 2012; Jackman et al., 2014), photostimulation was applied at 2 Hz. Electrical stimulation increased the promoter activity moderately at this frequency (Figures 4A,B; Ohmura et al., 2003). Similarly to the responses to the electrical stimulation, the luciferase signals started to increase within 2 h after 2 Hz photostimulation, and reached a peak at around 6 h (Figure 7B). The total signals and slope were significantly increased after the photostimulation (Figures 7C,D and Table 3). In contrast, 0.1 Hz photostimulation did not increase the luciferase signals with slight decrease after stimulation (Figures 7B–D and Table 3). Since only blue light exposure has been reported to affect the activity-dependent gene expression (Tyssowski and Gray, 2019), a photostimulation experiment was also conducted at 2 Hz without transfection of ChR2 (2 Hz cont in Figure 7). It is true that the luciferase signals were slightly increased after stimulation (Figures 7B–D and Table 3), but the extent was smaller than that in ChR2-transfected cortical slices (Figures 7E,F). These results indicate that excitatory synaptic inputs in cortical circuits are responsible for the modulation of Bdnf promoter activity.

**TABLE 3:** Brain-derived neurotrophic factor promoter activity in response to patterned optogenetic stimulation in mouse cortical neurons.

Stimulation	Cell #	Slice #	Before or after stimulation	Total signal (fold change × time [h])	Slope (fold change/time [h])
2 Hz	32	4	Before	1.7 ± 0.75	0.034 ± 0.015
			After	6.3 ± 1.2*	0.18 ± 0.021***
0.1 Hz	23	4	Before	1.9 ± 1.1	0.037 ± 0.024
			After	−2.6 ± 0.79**	0.010 ± 0.015
2 Hz wo ChR2	21	3	Before	1.8 ± 0.84	0.036 ± 0.014
			After	3.3 ± 0.99*	0.082 ± 0.016**

Asterisks indicate significant change (Mann-Whitney U test, *p < 0.05, **p < 0.01, ***p < 0.001).

## Discussion

In the present study, we demonstrated spatiotemporal regulation of Bdnf promoter activity in the cortex by pharmacological and physiological stimulation. Our results show that patterned stimuli differentially upregulate Bdnf promoter activity in individual upper layer neurons, *via* adequate increases of intracellular Ca^2+^ concentration. In addition, cortical cells showing similar activity dependence for gene expression are spatially localized. These results suggest that physiological stimuli differentially tune activity-dependent gene expression in individual cortical neurons *via* intracellular calcium signaling, which is controlled by intrinsic cortical cell properties and synaptic inputs.

### Modulation of Brain-Derived Neurotrophic Factor Promoter Activity by Patterned Stimulation

We demonstrated the precise time course and elevation of stimulus-induced Bdnf promoter activity in individual cortical cells. A remarkable aspect is that the levels of increase were modulated differentially by stimulus patterns. Indeed, distinct patterned activities have been found in the developing cortex, and they are thought to contribute to circuit refinement (Luhmann and Khazipov, 2018). Here, we showed that patterned stimulation such as GB, TB, and 20 Hz (long beta-oscillation) stimulation efficiently induced Bdnf expression, although high delta (2 Hz) and alpha waves (10 Hz) were only moderate inducers. These results suggest that neuronal activities, which are generated in the physiological situation, regulate molecular expression, which leads to the establishment of cortical wiring. In accordance with this view, recent studies have shown that patterned firing activity regulates guidance molecule expression (Mire et al., 2012; Nakashima et al., 2019). Moreover, the present optogenetic experiment demonstrated that patterned activation of pre-synaptic fibers increased Bdnf promoter activity, suggesting that physiological neuronal activity modulates the gene expression in individual cortical neurons *via* defined connections.

How do patterned activities differentially regulate the increase of Bdnf expression? The present results not only confirmed the necessity of the increase in intracellular Ca^2+^ concentration but also indicated that a transient increase is crucial for downstream molecular signaling. Ca^2+^-dependent transcriptional activity could be influenced by the temporally controlled Ca^2+^ concentration, which is differentially raised by stimulation patterns. Indeed, autophosphorylation of CaM kinase, which is critical for binding of transcription factors to Bdnf promoter IV, is regulated by Ca^2+^ stimulation at specific frequencies (Koninck and Schulman, 1998). The translocation of CREB-regulated transcription co-activator 1 (CRTC1) to the nucleus was also promoted by patterned stimulation (Kitagawa et al., 2017). It is likely that specific patterned neuronal firing, regardless of spontaneous or evoked activity, efficiently promotes Bdnf expression by increasing transient intracellular Ca^2+^ concentration in cortical cells and by upregulating the transcription together with cofactors. However, factors other than temporal changes in intracellular Ca^2+^ concentration may also be required for the upregulation of Bdnf expression, since temporal Ca^2+^ concentration changes and Bdnf expression were not completely proportional (Figure 6H).

Furthermore, we also found that patterned activity modulated the time courses of Bdnf promoter activity: 20 Hz and GB stimulation increased the promoter activity and generated a peak, but TB stimulation elicited gradual and continuous increases. Patterned stimulation may alter the temporal response by affecting the dynamics of transcription factors or their DNA binding. For instance, transcriptional activity of NF-κB, which also regulates Bdnf exon IV transcription, is modulated by the frequency of Ca^2+^ oscillation (Jin et al., 2006; Scharbrodt et al., 2009). Considering that NF-κB is translocated into the nucleus in response to Ca^2+^ stimulation, patterned stimulation may differentially regulate the temporal mobility of the transcription factor.

### Responsiveness of Individual Cortical Neurons

We also found different responsiveness in individual cortical neurons. The level of increase in promoter activity varied from cell to cell, even in uniform activation by KCl treatment, suggesting that activity-dependent responsiveness is intrinsically variable in cortical neurons (see “Materials and Methods”). In support of this view, diversity in activity-dependent promoter activity has also been reported in dissociated cortical cells from Bdnf-luc transgenic mice (Fukuchi et al., 2017). This may be due to different expression levels in calcium channels, cytoplasmic signaling molecules, and/or different phosphorylation levels of transcription factors, among cells (Giffin et al., 1991; Sala et al., 2000). These intrinsic biochemical properties may also contribute to the diversity of the proportion of responding cells in response to the stimulation patterns (Figure 4C). In fact, the Bdnf exon IV promoter contains three critical domains for calcium-dependent transcription, which is activated by calcium pathways that initiate from distinct calcium channels and calcium-stimulated kinases (Zheng et al., 2011). Considering that the various kinds of calcium channels may be differently expressed in each cortical neuron (Giffin et al., 1991), neuronal activities probably modify the proportion of responding cells by switching among types of activated calcium channels in response to activity patterns.

Regarding spatial distribution, cells with similar promoter activity were closely localized (Supplementary Figure 4), suggesting that cells with similar biochemical properties in their cytoplasmic molecular pathways tend to cluster. This spatial localization is in a very narrow range (0–30 μm), which is more restricted than the so-called columnar structures in the visual and somatosensory cortex (Levay et al., 1978; Petersen, 2007); rather, the localization is similar to the size of microcolumns (Kwan et al., 2012; Maruoka et al., 2017). Indeed, as cortical neurons originating from the same lineage have similar physiological properties and are spatially localized (Ohtsuki et al., 2012), these cells may have genetically similar properties. Moreover, this spatial profile was retained even by receiving effective physiological activities (Supplementary Figure 11 and Supplementary Table 4), which suggests that spatial arrangement is unaffected by neuronal network activation. However, which cell type exhibits the similarity activity-dependent increase remains unknown.

In summary, patterned stimulation differentially modulates the level of increase and time course of Bdnf promoter activity *via* transient changes in intracellular Ca^2+^ concentration and intrinsic biochemical properties within physiological neuronal circuits. Since BDNF released from cells promotes neurite outgrowth and branching of neighboring neurons (McAllister et al., 1996; Horch and Katz, 2002; Granseth et al., 2013), patterned neuronal activity may contribute to determination of the magnitude and time window in remodeling processes in neuronal wiring, and control local circuit formation by regulating Bdnf expression spatiotemporally.

## Materials and Methods

### Animals and Ethics Statement

All experiments were performed according to the guidelines established by the animal welfare committees of Osaka University and the Japan Neuroscience Society. Sprague–Dawley (SD) rats (Nihon–Dobutsu) and ICR mice (Japan SLC and CLEA Japan) were used in this study.

### Organotypic Slice Culture

Organotypic cortical slice cultures were prepared as described previously (Yamamoto et al., 1992). In brief, cortical slices were dissected from postnatal day (P) 1 rats or mice. The slices were placed on a membrane insertion (Millicell-CM; catalog no. PICMORG50; Millipore) coated with rat-tail collagen. The culture medium consisted of a 1:1 mixture of DMEM and Ham’s F-12 (Invitrogen) with several supplements (Yamamoto et al., 1992). The cultures were maintained at 37°C in an environment of humidified 95% air and 5% CO_2_.

### Construction of Luciferase Expression Vector

Based on the sequence information of rat Bdnf (NC_005102), the exon4 promoter region (387 bp, from -384 to +2) was amplified by PCR using KOD FX Neo DNA polymerase (TOYOBO) with a pair of primers (5′-GAATCCAGGTAGACAGCTTGGCAG-3′ and 5′-ACTGGGAGATTTCATGCTAGCTCG-3′). The template genome was obtained from rat tail. The PCR product was cloned into pGEM-T-Easy vector (Promega), and the promoter region was further amplified by PCR using primer pairs containing recognition sites of restriction enzymes, Nhe-I and Xho-I (5′-TCTAGCTAGCAAAGAAAGAAAGAAAAAAGAAAAG-3′ and 5′-ACTACTCGAGGTGGGAGTCCACGAGAG-3′). The PCR product was digested with the enzymes, and cloned into pGL3-basic vector (Promega), predigested with the same restriction enzymes.

### Local Cell Electroporation

To transfect the luciferase construct into upper layer cortical cells, electroporation with a glass microelectrode was performed after 2 days *in vitro* (DIV) as previously described (Uesaka et al., 2005). In brief, a mixture (0.5 μl) of Bdnf-luc plasmid (1.6 mg/ml) and pCAGGS-DsRed2 or pCAGGS-EGFP (0.4 mg/ml) in Hanks’ solution was applied to the surface of slices. Immediately afterward, electrical pulses (10 trains of 200 square pulses of 1-ms duration, 200 Hz, 450 mA) were delivered with a glass microelectrode (300 μm diameter). For calcium imaging, CAG-GCaMP6f vector (2.0 mg/ml) (Addgene) was transfected into cortical cells in the same way.

### Quantitative Reverse Transcription-PCR Analysis

After 1 or 2 weeks in culture, mRNAs were extracted from cortical explants that had been treated with KCl and TTX. After cDNA synthesis (Transcriptor First Strand cDNA Kit, Roche), gene expression was quantified by TaqMan Gene Expression Assay (Applied Biosystems). Rat GAPD was used as an endogenous control to normalize gene expression (Applied Biosystems). To amplify a specific sequence of Bdnf, a primer pair (5′-AGCGCGAATGTGTTAGTGGT-3′ and 5′-GCAATTGTTTGCCTCTTTTTCT-3′) and a universal probe were used.

### Real-Time Luciferase Assay

One day before the experiment, D-luciferin potassium salt (Wako) was added to the culture medium (final concentration, 0.1 mM). On the day of the experiment, cultured cortical slices were placed in an incubation chamber (Tokai Hit UK-A16U, 35°C, 5% CO_2_) on a microscope stage. After identifying DsRed2- or EGFP-positive cells, the luciferase signals were captured by an EMCCD camera (Andor, iXon3) attached to an upright microscope (Nikon, FN-S2N) through a 20x objective lens (NA, 0.5). The signal to noise ratio was increased by 4 × 4 binning, gain 1,000, and 5- or 10- min exposure.

After confirming that the luciferase signals were stable on the microscope stage, the recording was started. The signals were recorded for 3 h before pharmacological, electrophysiological, and optogenetic stimulation, and the slices were further observed for more than 12 h. The luciferase signals were taken at 30-min intervals.

### Pharmacological Treatments

To increase neuronal activity, a high potassium solution (final concentration, 25 mM) was applied to the culture medium approximately 3 h after starting live imaging. To suppress neuronal firing, TTX (final concentration, 100 nM; Seikagaku-Kogyo) was applied to the medium. To block synaptic transmission, D-AP5 (100 μM) (Tocris) and DNQX (20 μM) (Tocris) were applied prior to imaging. To suppress spontaneous activity, the concentrations of Mg^2+^ and Ca^2+^ were raised to 4 mM (Gustafsson et al., 1987). Picrotoxin (final concentration, 100 μM; nacalai tesque) was also applied to suppress the effect of inhibitory neurons. To block the increase in intracellular Ca^2+^ concentration, D-AP5 and nifedipine (10 μM) (Tocris) were added to the culture medium prior to imaging.

### Electrophysiological Stimulation

To apply electrical stimulation to cortical cells, we constructed hand-made electrode dishes, which are composed of a pair of platinum electrodes embedded in the bottom of culture dishes. One day before the experiment, the electrodes were inserted into cortical slices away from the pGL3-bdnf vector-transfected cells. For applying patterned activity, the electrodes were connected to an isolator (Bak Electronics, BSI-2), which was controlled by a programmable stimulator (A.M.P.I, Master-8). Various stimulation pulses (0.1 Hz for 20 min, 2 Hz for 10 min, 10 Hz for 2 min, 20 Hz for 1 min, 60 Hz for 20 s, GB for 10 min, and TB for 1 min) were then delivered to the slices (amplitude, 300 μA; duration 1 ms). GB and TB consist of a 60-Hz burst for 100 ms at 0.33 Hz and a 100-Hz burst for 30 ms at 5 Hz, respectively. A total of 1,200 pulses were applied for each frequency, and the chunk of electrical pulses was repeated every 20 min up to seven times.

### *In utero* Electroporation

*In utero* electroporation was performed to express CAGGS-hChR2(H134R)-EYFP (ChR2-EYFP) in a large number of upper layer neurons. As it was very difficult to perform the gene transfer technique in rats, mice were used in this experiment. Pregnant mice at E15 were deeply anesthetized with isoflurane. The abdomen was surgically opened without opening the uterus itself. ChR2-EYFP (2 μg/μl) in PBS was injected into one cerebral ventricle. A tweezers-type platinum electrode was positioned beside the uterus, and square pulses (40 V; 50 ms) were delivered five times with electroporator (CUY20; BEX). After electroporation, embryos were allowed to develop until birth.

### Optogenetic Stimulation

To apply stimulation to cortical cells, an optogenetic technique was also utilized. For this, a solid-state illuminator (475 nm peak wavelength; maximal power: 20 mW; Lumencor SPECTRA, Lumencor) was controlled by the programmable stimulator. The light intensity was 4.8 mW/mm^2^, which was enough to evoke the neuronal activity. Blue light stimulation (50 ms) was applied with various frequencies (0.1–2 Hz) through a 20× objective lens. The stimulation continued for 5 min and was repeated every 30 min up to five times.

### Immunohistochemistry

After the real-time luciferase assay, the slices were fixed for several hours with 4% paraformaldehyde in 0.1 M PBS. The slices were incubated at 4°C overnight with rabbit anti-Cux1 (1:250; Santa Cruz Biotechnology) and rat anti-GFP (1:1,000; nacalai tesque). After extensive washes, the signals were visualized with Alexa 488-conjugated anti-rat IgG (1:500; Invitrogen) and Cy5-conjugated anti-rabbit IgG (1:250; Jackson ImmunoResearch). The samples were embedded with 80% glycerol containing DAPI and DABCO, and observed by confocal microscopy through a 20× objective lens (Leica, TCS-SP5).

### Calcium Imaging

Calcium imaging was carried out using GCaMP6f or Oregon Green 488 BAPTA-1 (Invitrogen) (OGB-1). A CAG-GCaMP6f vector (Addgene) was electroporated sparsely to cortical neurons in the slice culture at 2 DIV (see above). Alternatively, 4 μM OGB-1 including 0.01% Pluronic F-127 and 0.005% Cremophor EL were added to the culture medium for 30 min. The culture was washed with Hanks’ solution before imaging.

In both cases, calcium imaging was performed in the chamber on the microscope. Excitation light (475 μm wavelength) was applied to the observed area with the solid-state illuminator through 20× objective lens. Images were captured by an EMCCD camera at 4 Hz (exposure time 100 ms; binning 2 × 2; gain 300). To measure stimulus-induced calcium signals, the baseline (F0) was calculated by averaging the signals prior to stimulation, and stimulus-evoked changes (ΔF) divided by F0 were determined.

To evaluate the increase levels for various stimulation patterns, stimulation with 0.1–60 Hz, GB, and TB burst was applied to the same cortical slices, and ΔF/F0 values were measured for a 0.4 × 0.4 mm area in the upper layers. The integral value of the ΔF/F0 values during 30 s stimulation period was then calculated for each stimulation pattern. This value for each stimulation was normalized by maximum value within all stimuli in each slice.

### Recording of Spontaneous Activity

To examine spontaneous firing in cortical slices, extracellular recording was performed on multi-electrode dishes (interpolar distance, 0.3 mm), as described previously (Uesaka et al., 2005). In brief, the extracellular potentials were amplified and stored in a hard disk after digitization. Experiments were performed in the presence of 4 mM Mg^2+^ and Ca^2+^ and 100 μM picrotoxin.

### Image Processing and Analysis of Temporal Properties

Luciferase signals were analyzed with image processing software (ImageJ Fiji). First, the region of interest was drawn within the cell body of each luciferase-positive cell, and the mean intensity was measured at every time point. The background signal outside the cell body was subtracted from the mean intensity. The subtracted value was smoothened by calculating the weighted moving average, and was defined as the signal intensity (L) at a given time point.

A baseline value (Lo) was calculated by averaging the signal intensities for 3 h prior to pharmacological or physiological stimulation. The ratio L/Lo was referred to as the “luciferase signal” to represent temporal changes of the luciferase signal. As the light intensity generated by the single luciferase plasmid is assumed to be summation of the baseline promoter activity (Po) and activity-dependent promoter activity after stimulation (P) (Choi et al., 2021), L can be represented as N × (Po + P) given that a cell is transfected with N plasmids. As the measured L increased approximately linearly after stimulation, P is A × t in the rising phase (A, constant; t, time after stimulation).

Hence, L/Lo = N ∗ (Po + A ∗ t)/N ∗ Po = 1 + (A/Po) ∗ t

Thus, the “luciferase signal” should not be affected by N but proportional to A/Po, which is considered to be a unique value for individual cells.

For the pharmacological treatments, the peak and lowest amplitudes were defined as the maximum and minimum values after stimulation, respectively. The total signal was calculated by integrating the signals over time after stimulation. The slope was determined as the maximum inclination for 6 h after stimulation.

In the stimulation experiments, the luciferase signal gradually increased even in the baseline period, which may be due to picrotoxin in the culture medium. Therefore, the total signals and slopes were compared before and after stimulation to assess the effects of patterned activity on Bdnf promoter activity. The total signals after the stimulation were calculated by integrating the signals over time. The total signal before stimulation was calculated during the baseline period and extrapolated. The slopes were defined as described above. The percentage of responding cells was determined by calculating the percentage of cells exhibiting signals exceeding the threshold, which was four times the standard deviation of the signals before the stimulation. The extent of sustained increase was calculated by subtracting the signal at 6 h from that at 12 h, and this value was normalized by the signal at 12 h.

### Analysis of Spatial Properties

For analysis of the spatial distribution of the cortical cells, the difference in the peak amplitude or the total signal between each cell pair was calculated. Cell pairs with a difference less than one-fourth of the maximal difference were defined as similar pairs, whereas those with a larger difference were defined as “different pairs”. Images of the analyzed neurons in fixed cortical explants were captured by confocal microscopy and the intercellular distance was measured by imageJ Fiji. The ratio of similar pairs was plotted in a histogram against the distance.

## Data Availability Statement

The raw data supporting the conclusions of this article will be made available by the authors, without undue reservation.

## Ethics Statement

The animal study was reviewed and approved by the animal welfare committees of Osaka University and the Japan Neuroscience Society.

## Author Contributions

YM performed all the experiments, analysis, and wrote the manuscript. NY performed a part of the experiments and wrote the manuscript. Both authors contributed to the article and approved the submitted version.

## Conflict of Interest

The authors declare that the research was conducted in the absence of any commercial or financial relationships that could be construed as a potential conflict of interest.

## Publisher’s Note

All claims expressed in this article are solely those of the authors and do not necessarily represent those of their affiliated organizations, or those of the publisher, the editors and the reviewers. Any product that may be evaluated in this article, or claim that may be made by its manufacturer, is not guaranteed or endorsed by the publisher.

## References

[B1] Ackman J. B. Crair M. C. (2014). Role of emergent neural activity in visual map development James. *Curr. Opin. Neurobiol.* 24 166–175. 10.1016/j.conb.2013.11.011 24492092 PMC3957181

[B2] Aid T. Kazantseva A. Piirsoo M. Palm K. Timmusk T. (2007). Mouse and rat BDNF gene structure and expression revisited. *J. Neurosci. Res.* 85 525–535. 10.1002/jnr.21139 17149751 PMC1878509

[B3] Cabelli R. Hohn A. Shatz C. (1995). Inhibition of ocular dominance column formation by infusion of NT-4/5 or BDNF. *Science* 267 1662–1666. 7886458 10.1126/science.7886458

[B4] Callaway E. M. Katz L. C. (1990). Emergence and refinement of clustered horizontal connections in cat striate cortex. *J. Neurosci.* 10 1134–1153. 10.1523/jneurosci.10-04-01134.1990 2329372 PMC6570203

[B5] Castren E. Zafra F. Thoenen H. Lindholm D. (1992). Light regulates expression of brain-derived neurotrophic factor mRNA in rat visual cortex. *Proc. Natl. Acad. Sci. U.S.A.* 89 9444–9448. 10.1073/pnas.89.20.9444 1409655 PMC50148

[B6] Chen W. G. West A. E. Tao X. Corfas G. Szentirmay M. N. Sawadogo M. (2003). Upstream stimulatory factors are mediators of Ca2+-responsive transcription in neurons. *J. Neurosci.* 23 2572–2581. 10.1523/jneurosci.23-07-02572.2003 12684442 PMC6742056

[B7] Choi J. Lysakovskaia K. Stik G. Demel C. Söding J. Tian T. V. (2021). Evidence for additive and synergistic action of mammalian enhancers during cell fate determination. *Elife* 10:e65381. 10.7554/eLife.65381 33770473 PMC8004103

[B8] Cruikshank S. J. Urabe H. Nurmikko A. V. Connors B. W. (2010). Pathway-specific feedforward circuits between thalamus and neocortex revealed by selective optical stimulation of axons. *Neuron* 65 230–245. 10.1016/j.neuron.2009.12.025 20152129 PMC2826223

[B9] Feldman D. E. Brecht M. (2005). Map plasticity in somatosensory cortex. *Science* 310 810–815. 10.1126/science.1115807 16272113

[B10] Flavell S. W. Greenberg M. E. (2008). Signaling mechanisms linking neuronal activity to gene expression and plasticity of the nervous system. *Annu. Rev. Neurosci.* 31 563–590. 10.1146/annurev.neuro.31.060407.125631 18558867 PMC2728073

[B11] Fukuchi M. Izumi H. Mori H. Kiyama M. Otsuka S. Maki S. (2017). Visualizing changes in brain-derived neurotrophic factor (BDNF) expression using bioluminescence imaging in living mice. *Sci. Rep.* 7:4949. 10.1038/s41598-017-05297-x 28694523 PMC5504055

[B12] Giffin K. Solomon J. S. Burkhalter A. Nerbonne J. M. (1991). Differential expression of voltage-gated calcium channels in identified visual cortical neurons. *Neuron* 6 321–332. 10.1016/0896-6273(91)90242-R 1848078

[B13] Gilbert C. D. Wiesel T. N. (1979). Morphology and intracortical projections of functionally characterised neurones in the cat visual cortex. *Nature* 280:120. 10.1038/280252b0 552600

[B14] Gilbert C. D. Wiesel T. N. (1989). Columnar specificity of intrinsic horizontal and corticocortical connections in cat visual cortex. *J. Neurosci.* 9 2432–2442. 10.1523/jneurosci.09-07-02432.1989 2746337 PMC6569760

[B15] Granseth B. Fukushima Y. Sugo N. Lagnado L. Yamamoto N. (2013). Regulation of thalamocortical axon branching by BDNF and synaptic vesicle cycling. *Front. Neural Circuits* 7:202. 10.3389/fncir.2013.00202 24391549 PMC3868945

[B16] Greer P. L. Greenberg M. E. (2008). From synapse to nucleus: calcium-dependent gene transcription in the control of synapse development and function. *Neuron* 59 846–860. 10.1016/j.neuron.2008.09.002 18817726

[B17] Gustafsson B. Wigström H. Abraham W. C. Huang Y. Y. (1987). Long-term potentiation in the hippocampus using depolarizing current pulses as the conditioning stimulus to single volley synaptic potentials. *J. Neurosci.* 7 774–780. 10.1523/JNEUROSCI.07-03-00774.1987 2881989 PMC6569059

[B18] Hayano Y. Sasaki K. Ohmura N. Takemoto M. Maeda Y. Yamashita T. (2014). Netrin-4 regulates thalamocortical axon branching in an activity-dependent fashion. *Proc. Natl. Acad. Sci. U.S.A.* 111 15226–15231. 10.1073/pnas.1402095111 25288737 PMC4210296

[B19] Hensch T. K. (2005). Critical period plasticity in local cortical circuits. *Nat. Rev. Neurosci.* 6 877–888. 10.1038/nrn1787 16261181

[B20] Hong E. J. McCord A. E. Greenberg M. E. (2008). A biological function for the neuronal activity-dependent component of bdnf transcription in the development of cortical inhibition. *Neuron* 60 610–624. 10.1016/j.neuron.2008.09.024 19038219 PMC2873221

[B21] Horch H. W. Katz L. C. (2002). BDNF release from single cells elicits local dendritic growth in nearby neurons. *Nat. Neurosci.* 5 1177–1184. 12368805 10.1038/nn927

[B22] Horch H. W. Krüttgen A. Portbury S. D. Katz L. C. (1999). Destabilization of cortical dendrites and spines by BDNF. *Neuron* 23 353–364. 10.1016/S0896-6273(00)80785-0 10399940

[B23] Iwasato T. Datwani A. Wolf A. M. Nishiyama H. Taguchi Y. Tonegawa S. (2000). Cortex-restricted disruption of NMDAR1 impairs neuronal patterns in the barrel cortex. *Nature* 406 726–731. 10.1038/35021059 10963597 PMC3558691

[B24] Jackman S. L. Beneduce B. M. Drew I. R. Regehr W. G. (2014). Achieving high-frequency optical control of synaptic transmission. *J. Neurosci.* 34 7704–7714. 10.1523/JNEUROSCI.4694-13.2014 24872574 PMC4035530

[B25] Jeanneteau F. Deinhardt K. Miyoshi G. Bennett A. M. Chao V. (2010). The MAP kinase phosphatase, MKP-1, regulates BDNF-induced axon branching. *Nat. Neurosci.* 13 1373–1379. 10.1038/nn.2655 20935641 PMC2971689

[B26] Jin S. Tian D. Chen J. G. Zhu L. P. Liu S. Y. Wang D. X. (2006). Passive sensitization increases histamine-stimulated calcium signaling and NF-κB transcription activity in bronchial epithelial cells. *Acta Pharmacol. Sin.* 27 708–714. 10.1111/j.1745-7254.2006.00334.x 16723089

[B27] Katz L. C. Shatz C. J. (1996). Synaptic activity and the construction of cortical circuits. *Science* 274 1133–1138. 10.1126/science.274.5290.1133 8895456

[B28] Kilb W. Kirischuk S. Luhmann H. J. (2011). Electrical activity patterns and the functional maturation of the neocortex. *Eur. J. Neurosci.* 34 1677–1686. 10.1111/j.1460-9568.2011.07878.x 22103424

[B29] Kitagawa H. Sugo N. Morimatsu M. Arai Y. Yanagida T. Yamamoto N. (2017). Activity-dependent dynamics of the transcription factor of cAMP-response element binding protein in cortical neurons revealed by single-molecule imaging. *J. Neurosci.* 37 1–10. 10.1523/JNEUROSCI.0943-16.2016 28053025 PMC6705672

[B30] Koninck P. D. Schulman H. (1998). Sensitivity of CaM kinase II to the frequency of Ca 2+ oscillations. *Science* 279 227–230. 10.1126/science.279.5348.227 9422695

[B31] Kwan K. Y. Lam M. M. S. Johnson M. B. Dube U. Shim S. Rašin M. R. (2012). Species-dependent posttranscriptional regulation of NOS1 by FMRP in the developing cerebral cortex. *Cell* 149 899–911. 10.1016/j.cell.2012.02.060 22579290 PMC3351852

[B32] Lee P. R. Cohen J. E. Iacobas D. A. Iacobas S. Douglas Fields R. (2017). Gene networks activated by specific patterns of action potentials in dorsal root ganglia neurons. *Sci. Rep.* 7:43765. 10.1038/srep43765 28256583 PMC5335607

[B33] Levay S. Stryker M. P. Shatz C. J. (1978). Ocular dominance columns and their development in layer IV of the cat’s visual cortex: a quantitative study. *J. Comp. Neurol.* 179 223–244. 10.1002/cne.901790113 8980725

[B34] Luhmann H. J. Khazipov R. (2018). Neuronal activity patterns in the developing barrel cortex. *Neuroscience* 368 256–267. 10.1016/j.neuroscience.2017.05.025 28528963

[B35] Madabhushi R. Kim T. K. (2018). Emerging themes in neuronal activity-dependent gene expression. *Mol. Cell. Neurosci.* 87 27–34. 10.1016/j.mcn.2017.11.009 29254824 PMC5894330

[B36] Majdan M. Shatz C. J. (2006). Effects of visual experience on activity-dependent gene regulation in cortex. *Nat. Neurosci.* 9 650–659. 10.1038/nn1674 16582906

[B37] Maruoka H. Nakagawa N. Tsuruno S. Sakai S. Yoneda T. Hosoya T. (2017). Lattice system of functionally distinct cell types in the neocortex. *Science* 358 610–615. 10.1126/science.aam6125 29097542

[B38] Matsumoto N. Hoshiko M. Sugo N. Fukazawa Y. Yamamoto N. (2016). Synapse-dependent and independent mechanisms of thalamocortical axon branching are regulated by neuronal activity. *Dev. Neurobiol.* 76 323–336. 10.1002/dneu.22317 26061995

[B39] McAllister A. K. Katz L. C. Lo D. C. (1996). Neurotrophin regulation of cortical dendritic growth requires activity. *Neuron* 17 1057–1064. 10.1016/S0896-6273(00)80239-1 8982155

[B40] Minlebaev M. Ben-Ari Y. Khazipov R. (2007). Network mechanisms of spindle-burst oscillations in the neonatal rat barrel cortex in vivo. *J. Neurophysiol.* 97 692–700. 10.1152/jn.00759.2006 17093125

[B41] Mire E. Mezzera C. Leyva-Diáz E. Paternain A. V. Squarzoni P. Bluy L. (2012). Spontaneous activity regulates Robo1 transcription to mediate a switch in thalamocortical axon growth. *Nat. Neurosci.* 15 1134–1143. 10.1038/nn.3160 22772332

[B42] Mizuno H. Hirano T. Tagawa Y. (2007). Evidence for activity-dependent cortical wiring: formation of interhemispheric connections in neonatal mouse visual cortex requires projection neuron activity. *J. Neurosci.* 27 6760–6770. 10.1523/jneurosci.1215-07.2007 17581963 PMC6672694

[B43] Munz M. Gobert D. Schohl A. Poquérusse J. Podgorski K. Spratt P. (2014). Rapid hebbian axonal remodeling mediated by visual stimulation. *Science* 344 904–909. 10.1126/science.1251593 24855269

[B44] Nakashima A. Ihara N. Shigeta M. Kiyonari H. Ikegaya Y. Takeuchi H. (2019). Structured spike series specify gene expression patterns for olfactory circuit formation. *Science* 365:5030. 10.1126/science.aaw5030 31171707

[B45] Niblock M. M. Brunso-Bechtold J. K. Riddle D. R. (2000). Insulin-like growth factor I stimulates dendritic growth in primary somatosensory cortex. *J. Neurosci.* 20 4165–4176. 10.1523/jneurosci.20-11-04165.2000 10818152 PMC6772633

[B46] Niculescu D. Michaelsen-Preusse K. Güner Ü van Dorland R. Wierenga C. J. Lohmann C. (2018). A BDNF-mediated push-pull plasticity mechanism for synaptic clustering. *Cell Rep.* 24 2063–2074. 10.1016/j.celrep.2018.07.073 30134168

[B47] Ohmura T. Ming R. Yoshimura Y. Komatsu Y. (2003). Age and experience dependence of N-methyl-D-aspartate receptor-independent long-term potentiation in rat visual cortex. *Neurosci. Lett.* 341 95–98. 10.1016/S0304-3940(03)00170-8 12686374

[B48] Ohnami S. Endo M. Hirai S. Uesaka N. Hatanaka Y. Yamashita T. (2008). Role of RhoA in activity-dependent cortical axon branching. *J. Neurosci.* 28 9117–9121. 10.1523/JNEUROSCI.1731-08.2008 18784292 PMC6670927

[B49] Ohtsuki G. Nishiyama M. Yoshida T. Murakami T. Histed M. Lois C. (2012). Similarity of visual selectivity among clonally related neurons in visual cortex. *Neuron* 75 65–72. 10.1016/j.neuron.2012.05.023 22794261

[B50] Olsen S. R. Bortone D. S. Adesnik H. Scanziani M. (2012). Gain control by layer six in cortical circuits of vision. *Nature* 483 47–54. 10.1038/nature10835 22367547 PMC3636977

[B51] Park H. Poo M. M. (2013). Neurotrophin regulation of neural circuit development and function. *Nat. Rev. Neurosci.* 14 7–23. 10.1038/nrn3379 23254191

[B52] Petersen C. C. H. (2007). The functional organization of the barrel cortex. *Neuron* 56 339–355. 10.1016/j.neuron.2007.09.017 17964250

[B53] Pfenning A. R. Kim T. K. Spotts J. M. Hemberg M. Su D. West A. E. (2010). Genome-wide identification of calcium-response factor (CaRF) binding sites predicts a role in regulation of neuronal signaling pathways. *PLoS One* 5:e10870. 10.1371/journal.pone.0010870 20523734 PMC2877716

[B54] Pruunsild P. Sepp M. Orav E. Koppel I. Timmusk T. (2011). Identification of cis-elements and transcription factors regulating neuronal activity-dependent transcription of human BDNF gene. *J. Neurosci.* 31 3295–3308. 10.1523/JNEUROSCI.4540-10.2011 21368041 PMC6623925

[B55] Rocamora N. Welker E. Pascual M. Soriano E. (1996). Upregulation of BDNF mRNA expression in the barrel cortex of adult mice after sensory stimulation. *J. Neurosci.* 16 4411–4419. 10.1523/jneurosci.16-14-04411.1996 8699252 PMC6578867

[B56] Ruthazer E. S. Stryker M. P. (1996). The role of activity in the development of long-range horizontal connections in area 17 of the Ferret Edward. *J. Neurosci.* 16 7253–7269. 8929433 10.1523/JNEUROSCI.16-22-07253.1996 PMC6578943

[B57] Sakata K. Woo N. H. Martinowich K. Greene J. S. Schloesser R. J. Shen L. (2009). Critical role of promoter IV-driven BDNF transcription in GABAergic transmission and synaptic plasticity in the prefrontal cortex. *Proc. Natl. Acad. Sci. U.S.A.* 106 5942–5947. 10.1073/pnas.0811431106 19293383 PMC2667049

[B58] Sala C. Rudolph-Correia S. Sheng M. (2000). Developmentally regulated NMDA receptor-dependent dephosphorylation of cAMP response element-binding protein (CREB) in hippocampal neurons. *J. Neurosci.* 20 3529–3536. 10.1523/jneurosci.20-10-03529.2000 10804193 PMC6772703

[B59] Scharbrodt W. Abdallah Y. Kasseckert S. A. Gligorievski D. Piper H. M. Böker D. K. (2009). Cytosolic Ca2+ oscillations in human cerebrovascular endothelial cells after subarachnoid hemorrhage. *J. Cereb. Blood Flow Metab.* 29 57–65. 10.1038/jcbfm.2008.87 18698333

[B60] Shieh P. B. Hu S.-C. Bobb K. Timmusk T. Ghosh A. (1998). Identification of a signaling pathway involved in calcium regulation of BDNF expression. *Neuron* 20 727–740. 10.1016/S0896-6273(00)81011-9 9581764

[B61] Suárez R. Fenlon L. R. Marek R. Avitan L. Sah P. Goodhill G. J. (2014). Balanced interhemispheric cortical activity is required for correct targeting of the corpus callosum. *Neuron* 82 1289–1298. 10.1016/j.neuron.2014.04.040 24945772

[B62] Tao X. Finkbeiner S. Arnold D. B. Shaywitz A. J. Greenberg M. E. (1998). Ca2+influx regulates BDNF transcription by a CREB family transcription factor-dependent mechanism. *Neuron* 20 709–726. 10.1016/S0896-6273(00)81010-7 9581763

[B63] Tao X. West A. E. Chen W. G. Corfas G. Greenberg M. E. (2002). A calcium-responsive transcription factor, CaRF, that regulates neuronal activity-dependent expression of BDNF. *Neuron* 33 383–395. 10.1016/S0896-6273(01)00561-X 11832226

[B64] Tyssowski K. M. DeStefino N. R. Cho J. H. Dunn C. J. Poston R. G. Carty C. E. (2018). Different neuronal activity patterns induce different gene expression programs. *Neuron* 98 530–546.e11. 10.1016/j.neuron.2018.04.001 29681534 PMC5934296

[B65] Tyssowski K. M. Gray J. M. (2019). Blue light increases neuronal activity-regulated gene expression in the absence of optogenetic proteins. *eNeuro* 6 ENEURO.0085–19.2019. 10.1523/ENEURO.0085-19.2019 31444226 PMC6751372

[B66] Uesaka N. Hayano Y. Yamada A. Yamamoto N. (2007). Interplay between laminar specificity and activity-dependent mechanisms of thalamocortical axon branching. *J. Neurosci.* 27 5215–5223. 10.1523/jneurosci.4685-06.2007 17494708 PMC6672371

[B67] Uesaka N. Hirai S. Maruyama T. Ruthazer E. Yamamoto N. (2005). Activity dependence of cortical axon branch formation: a morphological and electrophysiological study using organotypic slice cultures. *J. Neurosci.* 25 1–9. 10.1523/jneurosci.3855-04.2005 15634761 PMC6725200

[B68] West A. E. Chen W. G. Dalva M. B. Dolmetsch R. E. Kornhauser J. M. Shaywitz A. J. (2001). Calcium regulation of neuronal gene expression. *Proc. Natl. Acad. Sci. U.S.A.* 98 11024–11031. 10.1073/pnas.191352298 11572963 PMC58677

[B69] West A. E. Griffith E. C. Greenberg M. E. (2002). Regulation of transcription factors by neuronal activity. *Nat. Rev. Neurosci.* 3 921–931. 10.1038/nrn987 12461549

[B70] Wirth M. J. Brün A. Grabert J. Patz S. Wahle P. (2003). Accelerated dendritic development of rat cortical pyramidal cells and interneurons after biolistic transfection with BDNF and NT4/5. *Development* 130 5827–5838. 10.1242/dev.00826 14573511

[B71] Yamada A. Uesaka N. Hayano Y. Tabata T. Kano M. Yamamoto N. (2010). Role of pre- and postsynaptic activity in thalamocortical axon branching. *Proc. Natl. Acad. Sci. U.S.A.* 107 7562–7567. 10.1073/pnas.0900613107 20368417 PMC2867758

[B72] Yamamoto N. Yamada K. Kurotani T. Toyama K. (1992). Laminar specificity of extrinsic cortical connections studied in coculture preparations. *Neuron* 9 217–228. 10.1016/0896-6273(92)90161-6 1497891

[B73] Yang J. W. Hanganu-Opatz I. L. Sun J. J. Luhmann H. J. (2009). Three patterns of oscillatory activity differentially synchronize developing neocortical networks in vivo. *J. Neurosci.* 29 9011–9025. 10.1523/JNEUROSCI.5646-08.2009 19605639 PMC6665441

[B74] Yoshimura Y. Dantzker J. L. M. Callaway E. M. (2005). Excitatory cortical neurons form fine-scale functional networks. *Nature* 433 868–873. 10.1038/nature03252 15729343

[B75] Zheng F. Zhou X. Luo Y. Xiao H. Wayman G. Wang H. (2011). Regulation of brain-derived neurotrophic factor exon IV transcription through calcium responsive elements in cortical neurons. *PLoS One* 6:e28441. 10.1371/journal.pone.0028441 22174809 PMC3235121

[B76] Zheng F. Zhou X. Moon C. Wang H. (2012). Regulation of brain-derived neurotrophic factor expression in neurons. *Int. J. Physiol. Pathophysiol. Pharmacol.* 4 188–200. 23320132 PMC3544221

